# In rod we trust–The evaluation of a virtual rod and frame test as a cybersickness screening instrument

**DOI:** 10.1371/journal.pone.0313313

**Published:** 2024-11-11

**Authors:** Judith Josupeit

**Affiliations:** Engineering Psychology and Applied Cognitive Research, Institute of Work, Organizational and Social Psychology, Technical University Dresden, Dresden, Germany; Universiti Malaya Fakulti Perubatan: University of Malaya Faculty of Medicine, MALAYSIA

## Abstract

Although Virtual Reality (VR) holds massive potential, its applicability still faces challenges because some individuals experience cybersickness. This phenomenon includes general discomfort, disorientation, and/or nausea, and it threatens not only a pleasant user experience but also the user’s safety. Thus, predicting a user’s susceptibility without relying on screening questionnaires that focus on past experiences, would enable more pleasant, safer VR experiences, especially for first-time users. Hence, the current study uses the participant’s controller input in a virtual Rod and Frame Test (RFT) as an effortlessly trackable performance measure. The RFT is an established method for measuring an individual’s sense of verticality in visually displaced fields. It has been used in the context of simulator sickness and cybersickness. In line with the literature and the subjective vertical mismatch theory, a lower visual dependency is expected to be correlated positively with cybersickness. To evaluate the potential of the RFT as a screening method for cybersickness, a cybersickness-inducing virtual environment (the City) was deployed. In total, data from 76 participants were eligible for the statistical analysis. The study finds a positive correlation between lower visual dependency and cybersickness, but only for the group that took the RFT after experiencing the City and only for the post-RFT cybersickness ratings. As cybersickness symptoms were VR environment-specific, the predictive validity of the RFT considering the VR-specific attributes is limited. Further, other studies attributed different working mechanisms to explain the connection between visual dependence and cybersickness with conflicting evidence. Although the RFT is not applicable as a cybersickness screening method, the effect sizes suggest that the RFT could serve as an additional objective assessment of the individuals’ current state during VR exposure. Future research should systematically explore interconnections between the various factors that contribute to cybersickness, pursuing the idea of open science for context sensitivity.

## 1. Introduction

Virtual Reality (VR) allows for an immersive and interactive experience [1] via stereoscopic images, head-based rendering, and controller input. The technology enhances motivation, especially through the use of gamification, achieving a user experience similar to game-play in professional contexts [2]. Further, it individualizes training through user-specific feedback [3] and increases efficiency in prototyping through virtual mock-ups [4, 5]. There are virtually no limits to the possible applications of VR.

However, not all users perceive VR as intended, which hinders inclusive access to this technology. Knowing a user’s reaction before the VR exposure would enable a targeted fit between the user and the application. This is not only relevant from a commercial perspective, but also for occupational safety, as users might experience cybersickness—a form of Visually Induced Motion Sickness (VIMS) in VR. The simplest working model attributes the genesis of VIMS to the fact that afferences seen do not match with the afferences “felt”, e.g., vestibular, proprioceptive, somatosensory [6]. Like other forms of VIMS, the symptom severity of cybersickness ranges from mild discomfort to disorientation, dizziness, and nausea [7]. Thus, to ensure a pleasant—and, more importantly, safe—user experience and prevent injuries, especially for first-time users, predictive screenings for cybersickness susceptibility are indicated. This problem should not be underestimated; 30–80% of first-time VR users are expected to experience cybersickness with varying intensity [8]. Considering the wide range of applications of VR, cybersickness not only limits opportunities for the leisure activities of susceptible individuals but also hinders educational opportunities and careers [9].

Screening is often implemented using questionnaires. Among these, the Motion Sickness Susceptibility Questionnaire [MSSQ; 10] is, as the name suggests, applied in the contexts of both (physical) motion sickness (MS) and VIMS. The MSSQ focuses on symptoms that physical movement induces: The questionnaire items are a differentiation of types of transport or entertainment on a scale from 0 to 3 regarding one’s personal history of MS. Nevertheless, the reliability is low because of conventional issues with self-reports, such as recalling errors or social desirability, and because the questions are conflicted with a lack of distinction between real (i.e., physical) and apparent (i.e., virtual) movement [11]. Thus, the extent of the relationship between VIMS and MS is debatable. Hence, the applicability of screening questionnaires focusing on physical movement to infer cybersickness susceptibility is limited. VIMS and MS differ in symptom facets, severity, and potentially in the underlying working mechanism [12]. For example, the established Intra-Modality Conflict Theory for MS attributes children’s higher MS susceptibility to an immature vestibular system [13, 14]. In VR, there is a paucity of studies with minors [15]. From an ethical perspective, the maturation of the vestibular system could be irrelevant, as manufacturers of Head-Mounted Displays (HMD) advise against children under the age of 12 using their products in their safety regulations [16–18]. In addition, various software- and hardware-related effects influence cybersickness, leading to varying application-dependent symptom patterns [12, 19, 20]. Hence, many established factors for screening and working mechanisms must be reassessed in the context of cybersickness by considering the software and hardware specifications of the VR application. For specific use cases, a self-made abbreviated version of the MSSQ [21] or the VIMSSQ–a VR-specific revision of the MSSQ [22, 23], are suggested for use, but to date, large sample size validations of these rationales are lacking. Further, these questionnaires are only useful for screening individuals with a history of (VI)MS or evidently immune individuals. Those individuals who do not have any history of (VI)MS because of limited experience with physical or virtual provocative motion scenarios are hard to include.

The current study therefore takes a different approach: To increase the reliability and prospective validity of screening methods over the use of questionnaires, performance measures could be an alternative. Performance measures do not require the participant to correctly understand and interpret normative questionnaire items; rather, they perform a task that requires standardized but translatable and multi-modally presentable instructions. Therefore, performance measures would also work in scenarios with diverse participants, for example, in various languages, cultures, cohorts, or contexts, and they would not require any previous experience. One easily accessible performance measure is a participant’s controller input. As the application must already internally process this input for interactions with the VR application, it can be sampled without any bothering additional equipment [24].

One simple test that could be suitable to screen for cybersickness susceptibility is the Rod and Frame Test (RFT). In fact, the setup is so simple that Witkin and Asch designed the first physical room-scale version as early as 1948. In a completely darkened room, a luminous rod surrounded by a square frame was presented. The apparatus allowed the experimenter to tilt the rod and the frame along the longitudinal axis separately for each trial. Participants were tasked with adjusting the rod to their sensed vertical regardless of the orientation of the frame. The RFT is thus a metric for the extent to which a visual displacement affects an individual’s perception of verticality: While the visual displacement strongly influences some participants in their verticality assessment, others are not perturbed. From the perspective of personality psychology, individuals’ differing performance in the RFT is understood as a cognitive style: The theory of field (in-)dependence interprets the differences in RFT performance as the ability to separate figures in an embedded context [25, 26]. While field dependent individuals tend to consider external cues—that is, the surrounding frame—for their estimation of verticality, field independent individuals rely mostly on internal cues like proprioception or vestibular input [27].

In 1968, Barrett and Thornton [28] bridged field (in-)dependence and VIMS when they used the RFT as a screening method for simulator sickness. Field independent participants were expected to have a higher awareness of conflicting cues, sensing no motion but seeing motion in the fixed-based simulator, which was state-of-the-art technology. In line with their hypothesis, a positive correlation between field independence and self-reported discomfort for the RFT trials, in which the participants were seated in an upright position, was found. The importance of a match between ‘sensed vertical’ comprised of visual, vestibular, and other proprioceptive information and ‘subjective vertical’, that is, the expected afference, was stressed further in the subjective vertical mismatch theory [29]. According to this theory, a mismatch between the sensed and subjective vertical results in the experience of MS.

In contrast to the above-mentioned studies, this study shifts the focus from military and transportation simulations to a gamified task aimed at the general population. Furthermore, more ecological validity is achieved by deploying a gamified visuospatial orientation task in room-scale VR. To evaluate the prospective validity and trait stability of the participants’ performance in the RFT, the order of the two VR applications was randomized. In line with Barrett and Thornton [28], it is hypothesized that field in-dependence should positively correlate with self-reported cybersickness. More specifically, participants with a higher awareness of their physical vertical (mostly vestibular and some proprioceptive information) should experience a larger mismatch, as their expectations are likelier to conflict with the artificiality of the environment. As long as the tracking fidelity is sufficient (e.g., no losses in frame rate or delayed head-based rendering) field dependent participants, who use the external visual information, should be less aware of any mismatches.

Several prerequisites should be tested to evaluate the validity of the RFT as a screening instrument. First, a manipulation check that compares symptom reports at Baseline and Post-VR will rule out falsely attributing VR-unrelated symptoms as cybersickness [30]. In addition, the influences of aggravated symptom reports introduced through demand characteristics can be mitigated by subtracting the Baseline symptom reports from Post-VR [31]. This rationale enables a distinction between cybersickness and VR-unrelated feelings of uneasiness. The first prerequisite hypothesis follows from this and postulates a significant increase in the cybersickness symptom reports when the Baseline is compared to Post-VR.

In addition, for the VR RFT to be used as a universally applicable screening instrument, there should be no order effect of the VR exposures on RFT performance [32]. Therefore, both mean difference and equivalence in RFT performance were tested, focusing on the impact of order. If the RFT performance differs depending on the order of the VR exposures, the applicability as a screening task would be limited, and field (in-)dependence should not be considered as a trait. Nevertheless, the RFT could still be useful for screening if the correlation of field (in-)dependence with cybersickness is more pronounced in the group that takes the RFT first. Hence, these participants can be seen as the inexperienced group, without any conflicting previous VR experience.

Moreover, the repeated VR exposures require sufficiently long breaks to avoid the carry-over effects of cybersickness. These effects could either be systematical, in the sense that the second Baseline symptom report is generally higher compared to the first suggesting ineffective lengths of breaks, or they could also be imbalanced in that one VR application could result in systematically longer-lasting symptom reports than the other. Regardless, to legitimate the use of baseline-corrected symptom reports of cybersickness [20, 30], a third prerequisite hypothesis is added: Breaks between the VR exposures are sufficient such that no statistical difference or practical relevant influence of the previous VR exposure or a VR-application-specific after-effect is present.

## 2. Methods

### 2.1 Sample

In total, 87 participants volunteered for this study. Of these, six participants had to be excluded from the final analysis due to visual impairments. Four others were excluded, because they experienced severe symptoms of cybersickness, and one more was excluded because of technical problems with the position tracking. Of the remaining 76 participants, 28 identified as males and 48 as females. Twenty-three participants required a visual aid. Forty-five participants stated that they had no previous experience, 15 reported having previous experience up to 30 min, 15 reported having at least 30 min but less than 5 hrs of experience, and one reported more than 5 hrs. The age of the participants ranged from 18 to 55 years (*M* = 26.17 years, *SD* = 7.38). Participants were recruited through notices and flyers on campus and the participants’ data bank central experimental server of the Technical University Dresden (ORSEE3). As a safety requirement, participants needed to be of legal age (≥ 18 years) and lack a history of epilepsy or migraine. To control for effects of uneasiness that could refer to VR-unrelated visual effects pregnant participants and those with non-correctable visual impairments were excluded. Participants with visual impairments were asked to bring contact lenses if they had heavy-rimmed glasses that did not fit under the HMD. Participants were instructed to refrain from eating 2 hrs before their session. The study procedures conformed with the ethical guidelines of the Declaration of Helsinki and gained ethical approval beforehand from the local ethics committee of the Technical University Dresden (EK530122019).

### 2.2 Apparatus and measured variables

The study was conducted in the facilities of the Technical University Dresden from 21 November 2022 to 3 February 2023. The computer installed to render and display the virtual environment was custom-built with an Intel Core i7-9700K processor and an NVIDIA GeForce RTX 2070 graphic controller. The HMD was an HTC Vive (HTC 2018) with a dual AMOLED 3.6” diagonal display, a resolution of 1080 x 1200 px per eye, and a maximum refresh rate of 90 Hz. The experimental VR applications were custom-built, and the corresponding C#-code can be found on GitHub and runs in Unity (v2019.1.11f1.). The assets used were the Steam VR asset for custom key bindings of the HTC Vive controller and the Winridge City for prefab game objects. Moreover, all interactable objects were custom-built. In addition to the VR equipment, the experimenter could manually control the VR application by using a keyboard with all function-assigned keys explicitly labeled. A metadata log file sampled with approximately 60 Hz, the predefined refresh rate in the built Unity application. The log file contained the head positions and the performance measures, that is the controller input. Additionally, for the RFT, the rod rotations and constellations to the frame were recorded, while for the cybersickness-inducing gamified visuospatial orientation VR environment (the City), the collected checkpoints were counted. The HTC Vive infrared lighthouses, which enable the tracking of the HMD and the controller, were securely installed diagonally across the room, 3.24 m (lighthouse B) and 3.26 m (lighthouse C) from the participant at a height of 2.47 m (lighthouse B) and 2.55 m (lighthouse C). This led to a virtual room of 3.42 x 5.42 m. The participants were instructed to stand in a designated position in the calibrated area to standardize the position and viewing angle for the virtual RFT. The City allowed for slight movement but primarily constrained movement along the participant’s yaw axis.

The demographic questions were assessed via an online questionnaire (LimeSurvey) and displayed on a tablet (Samsung Galaxy Tab A10.1). The demographic questionnaire contained items regarding gender, age, the need for visual correction, the factor of previous experience on a categorical scale, and previous experience with studies on visuospatial orientation.

To check normal visual acuity, the Freiburg Visual Acuity Test (FrACT, v 3.10.5) was applied with the exclusion criterion of logMar < 0 [33, 34]. The FrACT was displayed on a second Lenovo Thinkpad E570 laptop with a 15.6” screen diagonal and an observer distance of 2.4 m (max. possible DecVA of 2.38). A USB numeric keypad with the Landolt-C orientations printed on the respective numbers was used to collect the participants’ entries. Additionally, as VR uses stereoscopic images stereo acuity was tested with the Random Dot Stereo Acuity Test (Vision Assessment Corporation, IL USA, 2007). Participants who had insufficient stereo acuity (i.e., less than borderline), were excluded to prevent confounding symptom reports, for example, eyestrain [35].

The questionnaires chosen to assess cybersickness were the Simulator Sickness Questionnaire [SSQ; 36] and the Misery Scale [MISC; 37]. The SSQ is by far the most widely applied symptom questionnaire not only in the field of simulator sickness but also cybersickness. It contains 16 symptoms on a scale from 0 (none) to 3 (severe). The MISC is a single-item questionnaire, from 0 (no symptoms) to 10 (nausea) with verbal anchors, which may help provide for an equal understanding of the scale. A previously determined termination criterion (MISC ≥ 6) was monitored during VR with an assessment interval of 2 min for reasons of safety [38].

### 2.3 Procedures

**Fig 1:**
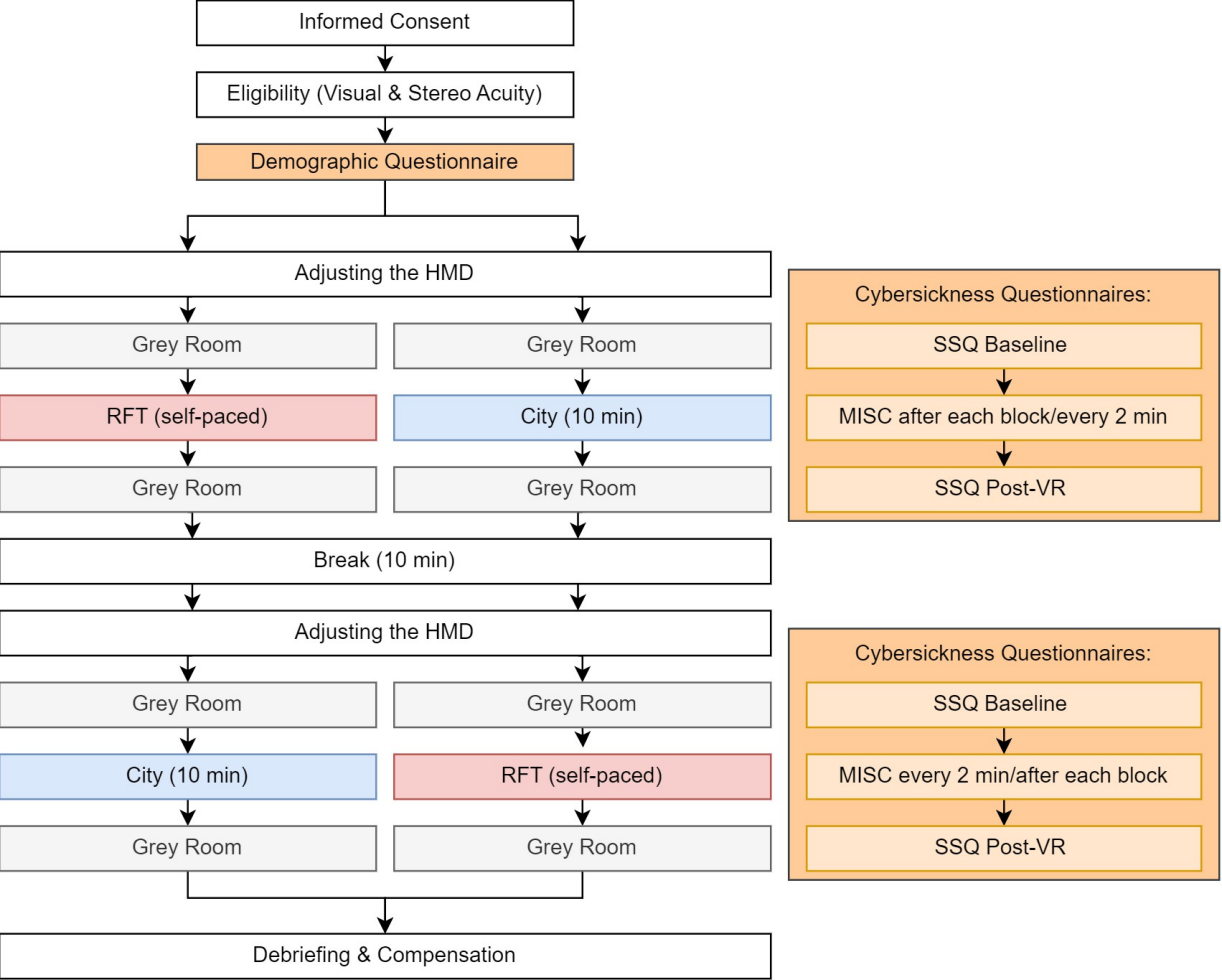
Procedure flowchart. The cybersickness questionnaire assessment rationale is presented in orange.

Fig 1 presents a flowchart of the experimental procedures.

After reading the information and giving written informed consent, tests for visual (stereo) acuity were conducted (FrACT and the Random Dot Stereo Acuity Test). If the participants required a visual aid, they were asked to wear it during the experiment.

When the participants passed the tests, they were given a tablet to complete the demographic questionnaire. The two questionnaires (SSQ and MISC), whose items were asked verbally during the experiment, were presented before the first assessment to the participants to clarify misunderstandings. Moreover, the handling and terminology of the controller’s components (trackpad and trigger) were explained. Thereafter, the participants were instructed to stand in a marked position in the calibrated area of the laboratory. The experimenter then adjusted the HMD. The experimenter asked the participant whether the image appeared blurry to them. Participants were allowed to adjust the interpupillary distance and eye relief until they perceived the image as sharply as possible, but this was determined only through the participant’s subjective estimate. Then, the participants were given the controller in their dominant hand and the Unity application was started. The application order was randomly assigned. In a VR waiting room, which was presented as a gray room with no additional lighting, the experimenter asked the participant for a baseline rating of the SSQ and the MISC. After participants affirmed their wish to continue, the experimenter explained the task of the corresponding application. While the participants performed their task, the experimenter monitored their behavior and performance in VR. To enable performance monitoring, the image of the HMD’s left lens was streamed on the display of the rendering computer. If necessary, the experimenter gave additional clarifying instructions, for example, if the functionality of the controller was misunderstood. This rationale also served a safety purpose in case the participant showed signs of severe discomfort.

**Fig 2:**
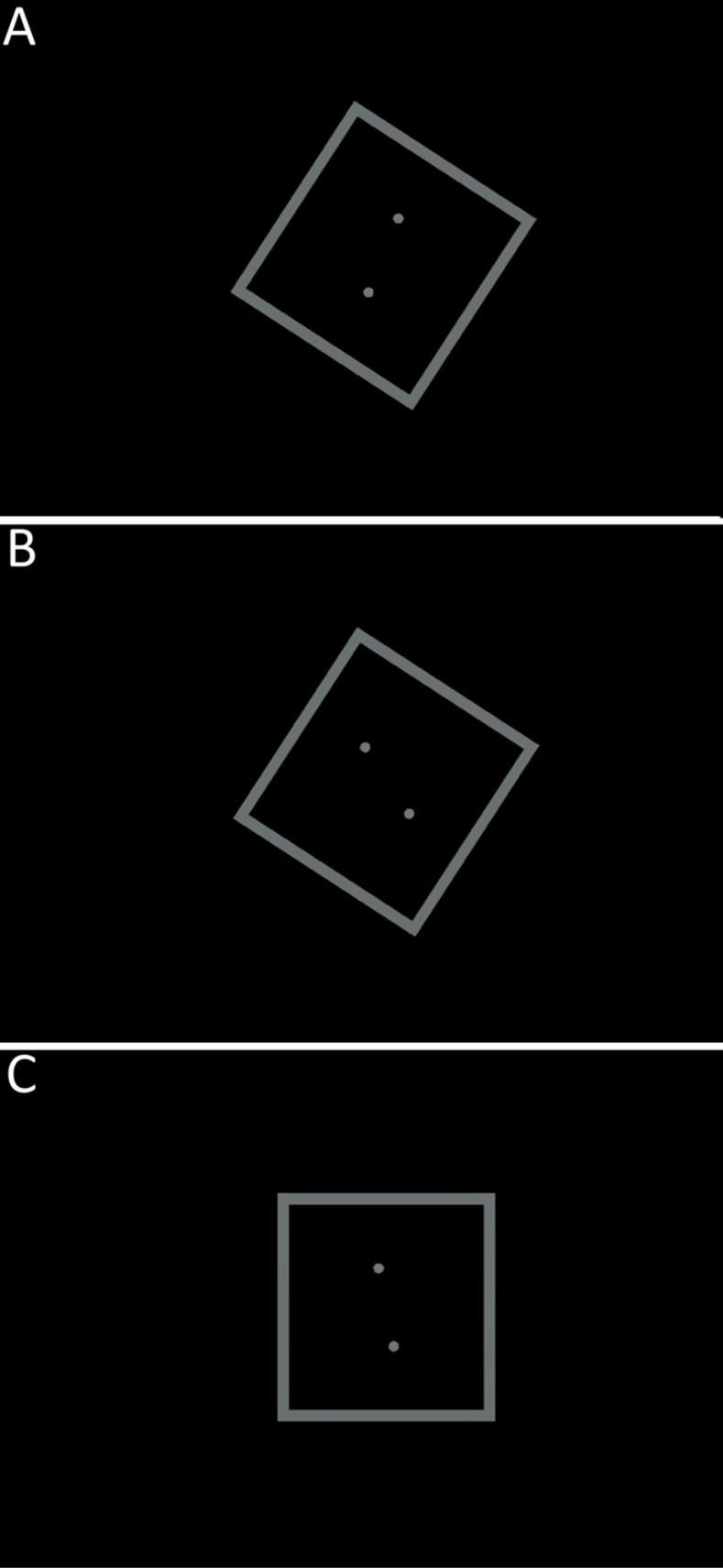
In-game screenshots of the RFT. Containing the following constellations: A) Compatible trial (tilt of the rod 22° and frame 33°), B) Incompatible trial (tilt of the rod -22° and frame 33°) and, C) Neutral trial (tilt of the rod -11° and frame 0°).

The next two paragraphs focus on the experimental tasks in the two applications: RFT and the City. For the former, during the RFT application, the participant was instructed to stand as still as possible to reduce any perspective artifacts by caused head rotation. This small detail of the participant’s posture is important for the classification results: Previous studies have used a seated RFT exposure [27, 28, 32, 39]. However, to replicate the work of Witkin and Asch [25], who used a room-filling RFT apparatus and a standing posture, a standing VR setup was used. This setup stresses the proprioceptive and vestibular parts of the subjective verticality assessment. Further, the setup is comparable to that of the City. Hence, this rationale also addresses the fidelity between the two applications [28, 40]. The RFT consisted of 36 randomized trials and two test trials that ensured understanding of the task and handling of the controller (see Fig 2). These test trials were ignored in the data analysis. The frame was tilted along the roll-axis of the Unity coordinate system in three manifestations (either—or +33° or 0°); the rotation of the rod had four manifestations (- or + 22° and—or +11°). This led to 12 different constellations, which were tested three times to ensure reliable results [39]. The participant’s task in each trial was to adjust an imaginary rod—two dots that needed to be conflated—vertically. The rod was expressed by two dots instead of one constant line—to reduce the clues of verticality because a tilted line would have a toothed appearance due to limitations of the resolution in VR [41]. By pressing either the left or the right side of the trackpad, the rod turned along the roll axis in either a positive or negative direction with 0.001° per input signal called once each frame [on “Update”; 42]. As the RFT was fully self-paced, the participant could confirm the assessment in each trial and start a new trial by pressing the trigger of the controller. When the participant accidentally pressed the trigger, the experimenter could reset the trial by pressing the escape button on the keyboard. After one set of constellations,—12 trials, excluding the two test trials—the gray waiting room was displayed to allow the experimenter to assess the MISC during the VR exposure. After the last trial, the waiting room was displayed again to conduct the post-VR assessment of the MISC and the SSQ. This part of the study lasted approximately 20 min, including the instructions. Since the participant self-paced clicking through the RFT application, durations for the RFT task, including only the performance for the 36 analyzed trials, varied from 2.87 to 13.00 min (*M* = 7.00, *SD* = 2.20).

**Fig 3:**
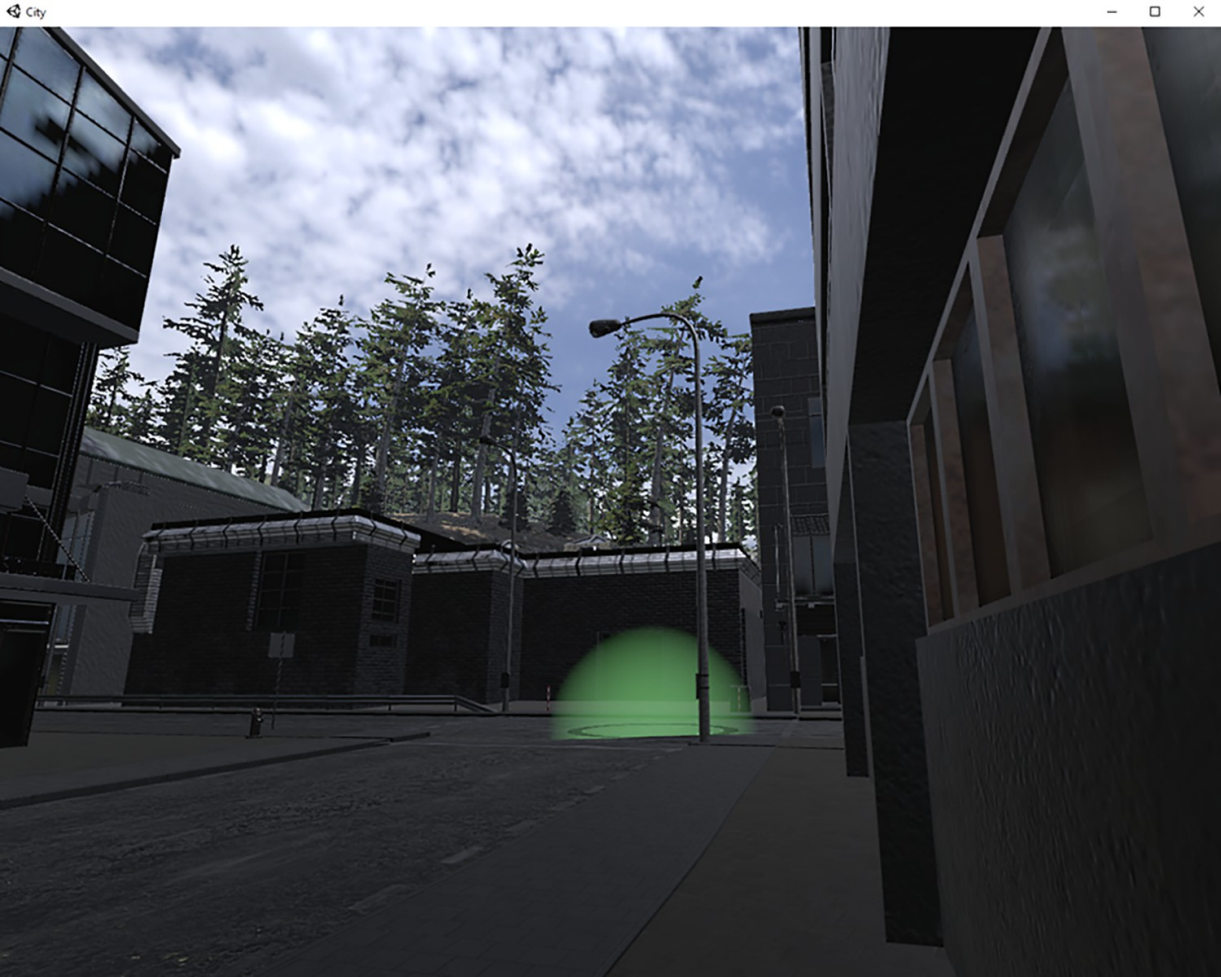
In-game screenshot of the city. Monocular display (left-lens) a virtual checkpoint (green) in the center.

In contrast to the constrained body posture in the RFT, the City application allowed participants to rotate themselves along their yaw axis. Using the trackpad, participants could navigate freely. The participants were told that the purpose of this environment was to test visuospatial orientation. To increase the ecological validity, gamification was implemented: The participant was tasked with finding as many virtual checkpoints as possible (maximum 11 checkpoints; *M* = 7.57, *SD* = 1.84.; range 1 to 10). The checkpoints were illustrated as green rings with a green glowing semi-circle in the center (see Fig 3). After the participant virtually walked through a checkpoint, the checkpoint disappeared and was logged automatically in the meta-data file. For an efficient search strategy, participants were advised to avoid reusing the same route multiple times. To reduce some unpleasant side effects, comfort functions such as teleportation are commonly used in game development instead of continuous locomotion [43]. As this environment was meant to provoke cybersickness, a custom-built continuous locomotion via the trackpad, like the magic carpet locomotion [44], was implemented. The touch of the trackpad allowed longitudinal acceleration, a movement that has the highest impact on cybersickness [45], and releasing the trackpad allowed for a sudden full stop. To change the direction, the participant needed to rotate their head. Every two minutes, the well-being of the participant was verbally assessed with the MISC. After 10 min of VR exposure, the waiting room was displayed again and the MISC and the SSQ were asked post-VR.

Regardless of the starting condition, a 10-minute break followed, during which the HMD was removed and the participants were offered a beverage and sweets. Although short breaks may not be sufficient to fully alleviate carry-over effects [46], the experimenter needed time to modify the experimental setup, and the standardized break durations were used to reduce the likelihood of carry-over effects. Thereafter, the second application started and followed the same procedures as mentioned above. After the second VR exposure, the participants were debriefed and compensated with either € 10 or course credit before they were dismissed.

### 2.4. Design

The dependent variable, cybersickness was repeatedly measured over time, the SSQ four times and the MISC ten times. To control for VR-unrelated symptoms [30], the Δ-SSQ scores were calculated by subtracting the baseline from post-VR ratings [20, 47]. The dependent variable, RFT performance, deviance from perfect verticality or no rotation, was repeatedly measured in each constellation 3 times, which led to 36 iterations in total. Different indices were calculated: Firstly, the mean absolute deviation from the compatible and incompatible trials [26, 32, 48], as this indicator does not differentiate between the real frame dependency and a participant’s internal response consistency; secondly, a constant error that focuses on the participant’s tendency to select one side regardless of either frame rotation or the rod starting position was added [49]. The constant error is the average of all trials but the neutral ones, as these were not considered in previous evaluations of the scoring method. Thirdly, the frame effect was calculated by subtracting the constant error from the mean right-tilted frame condition. Lastly, to include the neutral condition, the frame influence was calculated by subtracting the mean angle error in the neutral condition from the mean angle error of the compatible and incompatible conditions [39].

To test the predictive power of a participant’s performance in the RFT for cybersickness ratings in the City, a within-subjects analysis was required. A G*Power 3.1.9.2. analysis estimated a sample size of 67, assuming a medium effect size ρ = .3 (ρ_0_ = 0) with an α-error probability of .05 and a power (1-β-error probability) of .8 [50, 51]. The data preprocessing, plotting, and analysis were conducted in R 4.3.2 [52]. Noteworthy packages for the frequentist inference were lme4 [53], effectsize [54], and MANOVA.RM [55].

## 3. Results

### 3.1. Data preprocessing

Before running inference statistics on the data, some data preprocessing was performed. The constellation of the rod rotation and the respective frame rotation were extracted, and the absolute time for each trial was calculated. The beginning of each trial was marked by the first registered trackpad input and the end of each trial was marked by the confirmation a participant gave by pulling the trigger. Moreover, to gain the positive and negative deviations from 0°, values that were larger than 180° were subtracted from 360°. Additionally, the sum of the checkpoints reached in the City was extracted by using the last integer of the checkpoint counter.

### 3.2 Descriptive statistics

In the upcoming paragraphs of the results section, different font styles are used for more clarity. Labels for factors of the statistical models are written in *Italics* in the running text, their levels are written with a capital letter, and the font Calibri Light is used for dependent variables.

**Fig 4:**
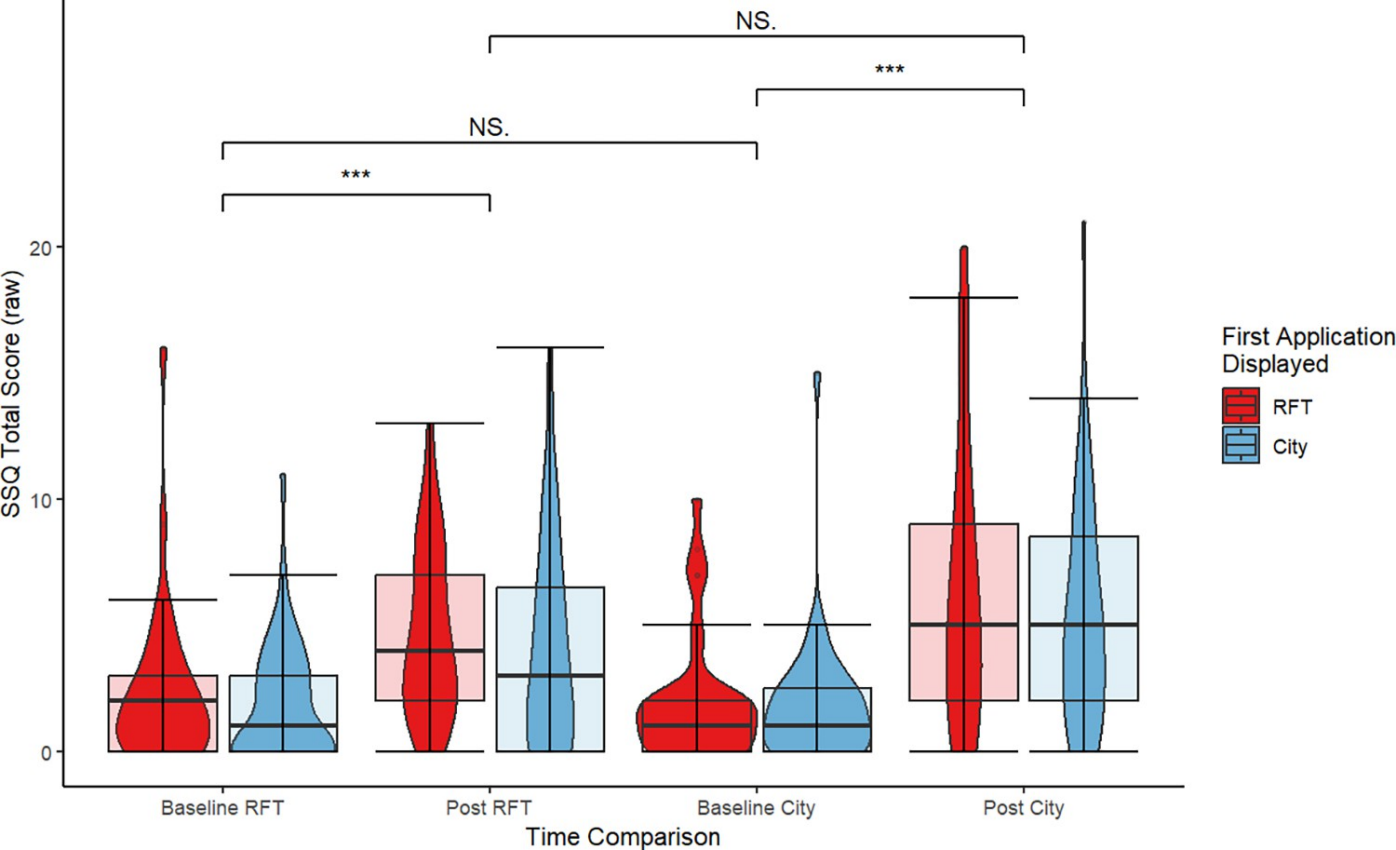
Comparison of baseline and Post-VR of self-reported cybersickness by *First Application Displayed*. Violin plots with overlayed boxplots α-level = .05.

**Fig 5:**
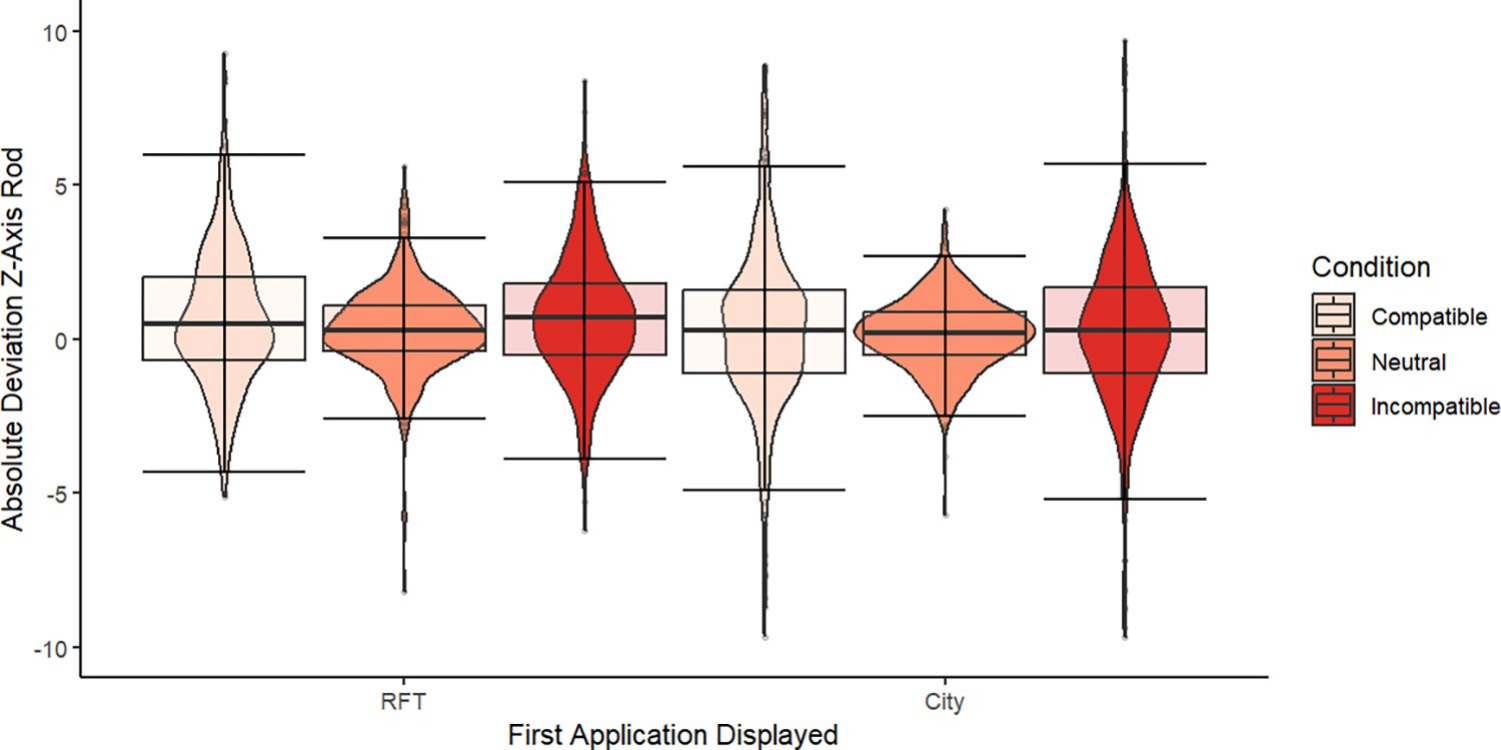
Performance measures of the RFT by *Condition* and *Application Order*. Violin plots with overlayed boxplots.

The medians and IQRs of the raw self-reported cybersickness and performance measures, are shown in Tables 1 and 2. The table facilitates the classification of the raw SSQ total score and the raw deviation from 0° of the rod in the RFT plotted in Figs 4 and 5, as it also includes the information regarding the *First Application Displayed*.

**Table 1:** Assorted descriptive statistics of the self-reported cybersickness.

Environment	Assessment Time	First Application Displayed	SSQ_total score (raw)_
RFT	Baseline	RFT	2 (3)
		City	1 (3)
	Post-VR	RFT	4 (5)
		City	3 (6.5)
City	Baseline	RFT	1 (2)
		City	1 (2.5)
	Post-VR	RFT	5 (7)
		City	5 (6.5)

The descriptive statistics display the median and the respective IQR in brackets for the non-weighted SSQ total scores in the Baseline and Post-VR comparison with the between-subjects factor *First Application Displayed* (RFT *N* = 33; City *N* = 43).

**Table 2:** Assorted descriptive statistics of the performance measure.

First Application Displayed	Condition	Deviation from 0°
RFT	C	0.451(2.7)
City	C	0.301 (2.8)
RFT	I	0.7 (2.4)
City	I	0.301 (2.8)
RFT	N	0.3 (1.5)
City	N	0.2 (1.4)

The mean and the respective standard deviation in brackets of the deviation from 0° along the vertical axis for the RFT performance measure are displayed for all three conditions (C = Compatible, I = Incompatible, N = Neutral); they are, also grouped by the between-subjects factor *First Application Displayed* (RFT *N* = 33; City *N* = 43).

The visualization (Fig 4) for the self-reported cybersickness deployed the non-weighted raw SSQ total score for a Baseline vs. Post-VR comparison. It is apparent that the SSQ data are right skewed but increase when comparing Baseline to Post-VR. The variance also increases with prolonged VR exposure. Regardless of the *First Application Displayed*, the City *Environment* seems a little more effective in inducing cybersickness, although the differences in Post-VR between SSQ_RFT_ and SSQ_City_ are low. The *First Application Displayed* does not seem to impact the reported symptoms.

For the RFT visualization (Fig 5), analogously, the raw deviation from 0° was used. In addition to the *First Application Displayed*, the three *Conditions* were respected in the visualization. Visual inspection suggests that the Neutral Condition resulted in the best performance compared to the other two conditions, regardless of the *First Application Displayed*. The distributions are almost equal for the Incompatible and Compatible Conditions. When considering the median, the participants who started with the RFT seem to perform a little worse in the Incompatible Condition. Although some extreme deviations from 0° were present for both *Conditions* (Incompatible and Compatible), most data are inside the error bar margins. Less than 1% (exactly 0.841%) of the detected outliers (total proportion of outliers 4.386%) were extreme. For the outlier identification, the boxplot method was used [56]. All data, including the extreme outliers, were kept, as this corresponds to the performance measures sampled. In particular, the internal response consistency would not be comparable between subjects if varying amounts of data had been used to calculate the indices. Further, it can be argued that, because robust tests were performed, the influence of the outliers can be evaluated as limited.

### 3.3. Inference statistics

As the first prerequisite hypothesis anticipates a significant difference between the Baseline rating and Post-VR on the self-reported cybersickness, only the factor *Environment* (levels RFT/City) with the repeated measures factor *Assessment Time* (levels Baseline/Post-VR) were considered for this analysis. The assumption of normality for the raw SSQ total score was tested with a Shapiro-Wilks test. As the visualizations already suggested, the tests revealed that the data were not normally distributed (all *W* ≤ 0.916, *p* < .001). Since the following analyses required the raw SSQ total score instead of transformations, alternatives to parametric tests were used, which is common practice in cybersickness research [57]. A resampling-based longitudinal equivalent to a repeated measures ANOVA with the factor *Environment* was calculated [55]. A significant main effect for the factor *Environment* (ATS(1, ∞) = 4.634, *p* = .031) revealed higher values in the City. Furthermore, for the factor *Assessment Time*, significantly higher values Post-VR were found (ATS(1, ∞) = 127.525, *p* < .001). Additionally, the interaction of the factors was significant (ATS(1, ∞) = 7.584, *p* = .006) with a smaller contrast between the *Assessment Times* for the *Environment* RFT.

For the second prerequisite hypothesis stated no influence of the *First Application Displayed* on the performance measures of the RFT. Hence, a linear mixed model with *First Application Displayed* as a fixed effect and *Condition* as a random slope for the effect of rod deviation from 0° was used. As fixed effects, the *Condition* and the *First Application Displayed* were included in the model. As random effects, an intercept for the *Condition* by-subject was included as a random slope. Visual inspection of the residual plots did not reveal any obvious deviations from homoscedasticity or normality. The random slope *Condition* affected the deviation from 0° (χ^2^(6) = 21.191, *p* = .002). P-values were obtained by likelihood ratio tests of the full model with the effect in question against the model without the effect of *Condition*. For a detailed overview of the estimators and the model, see Table 3. Additionally, equivalence testing for a $\Omega_{p}^{2}$ of .01—which would be a very small effect, according to Cohen [58]—was run for the model. It could be concluded that the null hypothesis for the *First Application Displayed* could be accepted $\Omega_{p}^{2}\approx 0$; CI 90%[0.00, 0.00], while the decision for the factor *Condition* was undecided $\Omega_{p}^{2}$ = 0.02; CI 90%[0.00, 0.06].

**Table 3:** Estimators of the linear mixed model for the performance measures.

Fixed Effects							
	Est/Beta	*SE*	95% CI			*t*	*p*
Intercept	0.221	0.266	-0.306–0.746			0.831	0.408
First Application Displayed	0.097	0.267	-0.430–0.625			0.362	0.718
Condition Incompatible	0.463	0.284	-0.100–1.026			1.633	0.107
Condition Neutral	-0.027	0.283	-0.586–0.533			-0.094	0.926
Random Effects							
			Variance	*SD*	Correlation		
Participant (Intercept)			2.111	1.453			
Condition: Incompatible (Intercept)			1.646	1.283	-0.61		
Condition: Neutral (Intercept)			1.623	1.274	-0.99		0.69
Residual			26.822	5.179			

Linear mixed model fit by maximum likelihood. The t-tests used Satterthwaite’s method. Confidence intervals were calculated using the Wald method.

Model equation: Deviation from 0° ~ First Application Displayed + Condition + (1 + Condition | Participant)

Further, the influence of the application order was tested in a linear mixed model with the *First Application Displayed* as a fixed effect and the *Environment* and *Assessment Time* as random slopes for the raw SSQ total scores. This analysis is a diversification of the first prerequisite hypothesis, as the between-subjects factor *First Application Displayed* was added. The models that included both random slopes had a significantly better fit than the respective null model, that is, without the respective factor (factor *Assessment Time*: χ^2^(4) = 140.313, *p* < .001; factor *First Application Displayed*: χ^2^(4) = 13.866, *p* = .008). The residual plots did not indicate any coarse deviations from homoscedasticity or normality. P-values were obtained by likelihood ratio tests of the full model with the effect in question against the model without the effect of *Environment*. Table 4 provides a summary of all estimators and the model. Additionally, equivalence testing for an equally small effect of $\Omega_{p}^{2}$ = .01 was run for the model. It could be concluded that the null hypothesis for the *First Application Displayed* could be accepted $\Omega_{p}^{2}\approx 0$; CI 90%[0.00, 0.00], while the decision for the factor *Environment* was undecided $\Omega_{p}^{2}$ = 0.04; CI 90%[0.00, 0.14] and was rejected for the factor *Assessment Time* $\Omega_{p}^{2}$ = 0.51; CI 90%[0.39, 0.60].

**Table 4:** Estimators of the linear mixed model for the self-reported cybersickness.

Fixed Effects								
	Est/Beta	SE	95% CI		*t*		*p*	
Intercept	5.284	0.553	4.20–6.37		9.555		< .001	
First Application Displayed	0.347	0.540	-0.72–1.41		0.642		0.522	
Environment	-0.632	0.293	-1.21– -0.05		-2.152		0.031	
Assessment Time	-3.092	0.314	-3.71 –-2.47		-9.867		< .001	
Random Effects								
			Variance	*SD*		Correlation		
Participant (Intercept)			15.891	3.986				
Environment: RFT (Intercept)			2.321	1.523		-0.51		
Assessment Time: Baseline (Intercept)			3.254	1.804		-1.00		0.47
Residual			4.226	2.056				

Linear mixed model fit by maximum likelihood. The t-tests used Satterthwaite’s method. Confidence intervals were calculated using the Wald method.

Model equation: SSQ _Total Score raw_ ~ First Application Displayed + Environment + Assessment Time + (1 + Environment + Assessment Time | Participant)

**Fig 6:**
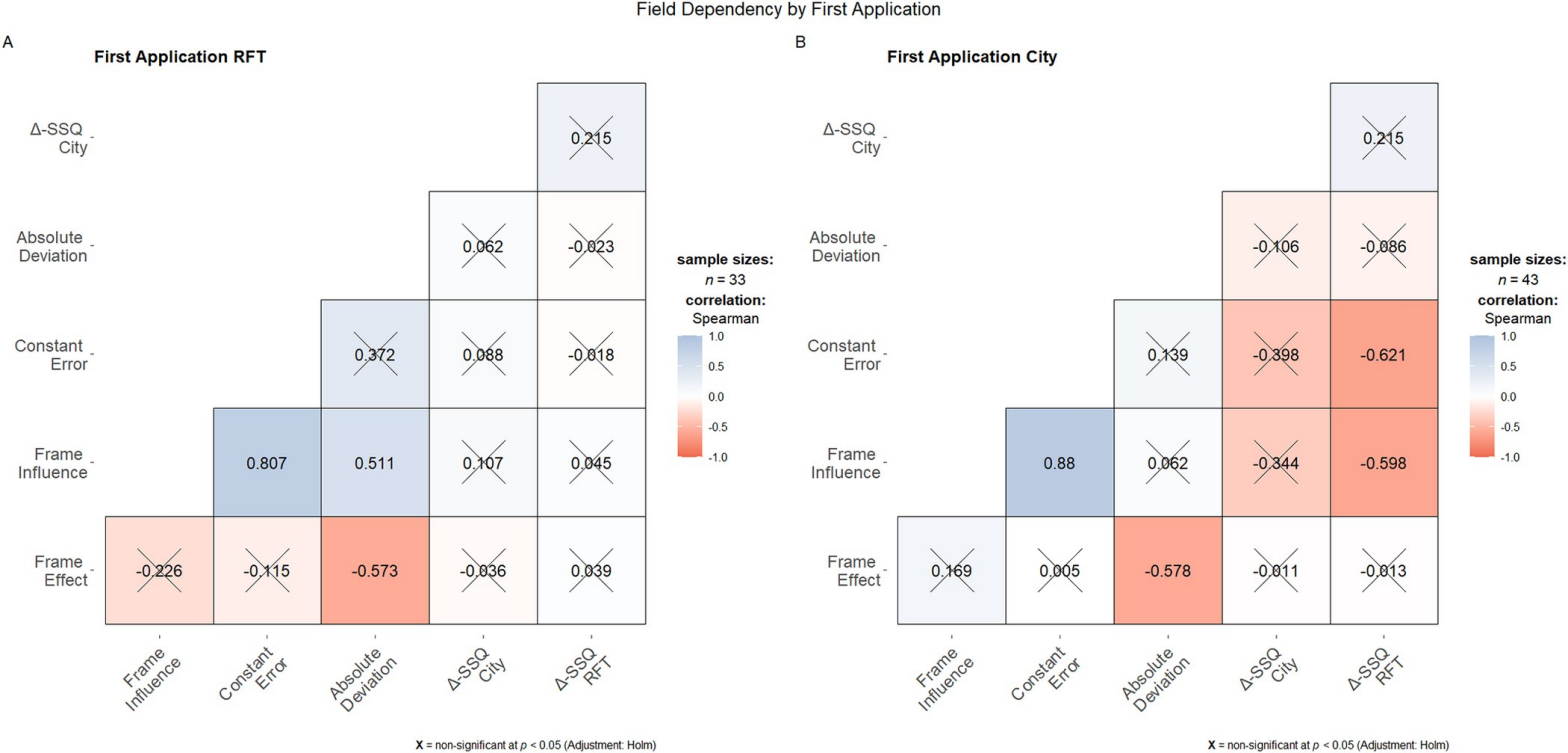
Correlogram of self-reported cybersickness and field dependence by *First Application Displayed*. Non-significant correlations at Holm-corrected α-level of .05.

The main hypothesis postulates a positive correlation of field independence and cybersickness. Cybersickness was operationalized as the Δ-SSQ ratings that were calculated by subtracting an individual’s baseline rating from the respective post-VR rating. For the operationalization of field independence different indices co-exist; thus, assorted indices based on previous validations [39, 48, 49] were used. As with the raw deviation from 0°, smaller numbers indicate higher field independence. The non-parametric Spearman’s rank correlations were computed to assess the relationship between field dependency and cybersickness grouped by the *First Application Displayed*. As an adjustment for multiple comparisons, Holm’s method was applied. For a detailed overview of all correlations see Fig 6. Through visual inspection, it becomes apparent that the correlation coefficients differ not only in strength but also in the direction of the correlation. While none of the indices correlate with any Δ-SSQ rating for the group that started with the RFT, the results for the group that started with the City show a pronounced negative correlation of frame influence (*ρ* = -0.598, n = 43, p < .001) and the participant’s constant error (*ρ* = -0.621, n = 43, p < .001) with the Δ-SSQ_RFT_. Interestingly, the positive correlations between the Δ-SSQs in both VR environments were small and non-significant (*First Application* RFT *ρ* = 0.215, n = 33, *p* ≈1, n.s.; *First Application* City *ρ* = 0.215, n = 43, *p* ≈1, n.s.).

## 4. Discussion

This study evaluated the prospective validity of the virtual RFT as a screening instrument for cybersickness. More specifically, it was hypothesized that a field independent participant would report higher cybersickness in an ecologically valid VR application (the City). The City was designed to induce cybersickness while addressing aspects of gamification. To test the universality of the RFT as a screening instrument, this study used the two VR applications in randomized order, which made it possible to control for influences of instantaneous previous VR experience. The hypothesis could not be confirmed, but one exception emerged: The two indices, constant error and the field dependency index, were significantly negatively correlated with the Δ-SSQ_RFT_ only for the group that started with the City.

As a manipulation check, higher symptom reports post-VR exposure compared to baseline were expected and were confirmed for both environments. In direct comparison, the reported symptoms were lower for the RFT compared to the City regardless of the application order. This was implicitly expected as the main purpose of the RFT was to assess field (in-)dependence, while the City’s main purpose was to induce cybersickness. Thus, the implemented features in the City seem to have worked as intended. Regarding SSQ items general discomfort, sweating, nausea, dizziness with the eyes open and closed, vertigo, and stomach awareness were rated higher in the City compared to the RFT. The findings are descriptively relatable to previous research that compared the items of the VRSQ—a VR-specific revision of the SSQ—in different environments [20]. However, Bonferroni-Holm corrected univariate comparisons of the items revealed that the global effect of the application (MATS (1,16) = 56.251, *p*_Bs_ < .001) was only attributable to the item nausea (*p* < .001). Further, on the aggregated level, no statistically significant correlation between the Δ-SSQ_RFT_ and the Δ-SSQ_City_ was found. Thus, the different applications not only invoked different symptom patterns and higher reports for the City, but the participants’ susceptibility was also individually different for both applications, which is an obstacle for universal screening instruments.

Moreover, it was tested whether the performance measures of the RFT were influenced by the order in which participants were tasked with the applications. The group that started with the City had at least 10 min of previous VR experience and was familiar with handling the controller. A more accurate estimation of verticality would not only be attributable to a different perceptual style but also to a greater familiarity with VR. However, by null hypothesis testing for the influence of the application order on the RFT raw scores, this argument was invalidated. Nevertheless, the previous familiarization with the controller might have influenced the performance between the groups. The self-paced task duration of the RFT was almost equal for both groups (first application City *M* = 7.09 min, *SD* = 2.15; RFT *M* = 6.88 min, *SD* = 2.30). Nevertheless, it was exploratively analyzed whether the frequency of stops in controller input relative to the individual’s absolute task completion time per trial was significantly different between the groups. The test for multivariate data found significantly shorter breaks for the group that started with the City (MATS(1,36) = 6.613, *p*_Bs_ = .012; first application City *M* = 0.515, *SD* = 0.532; RFT *M* = 0.568, *SD* = 0.528). This means the group that started with the City took fewer breaks in each trial although the task completion time was about the same.

Hence, although the task completion times are comparable for both groups, a thorough task performance for the group that started with the City could at least moderate the accuracy and therefore reliability of the RFT performance [59].

Interestingly, for the different conditions (i.e., compatible, incompatible, and neutral), null hypothesis testing was not rejected but rather undecided, which could be interpreted as either a relatively noisy measurement or an insufficient manipulation of the visually displaced field (i.e., the frame). As the fit of the model with the random slope condition was significantly better than the null model, calculating the indices for field independence was still justified. Nevertheless, this ambiguous effect must be carefully considered to avoid drawing false conclusions based on the interpretation of any further analyses. These results advocate for the use of null hypothesis testing in lieu of “classic” difference tests to estimate indeterminacy [60], even if the difference test is significant.

The influence of the application order on potential carry-over effects of self-reported cybersickness was further evaluated using equivalence tests. The model confirmed the null hypothesis for very small effects $\Omega_{p}^{2}$ = .01. This is interesting, as other studies have found diverging evidence for the sufficiency of short breaks (10 min), which would result in inflated baseline ratings for the second VR exposure [46, 61, 62]. The current results suggest that the standardized breaks of 10 min were effective in alleviating the symptoms, as the first and second baseline in both groups did not statistically or practically differ from one another. As the length of the breaks was almost equal to the VR exposures, further validations of the effectiveness of breaks in the dependency of the length of a VR exposure could help validate this rule of thumb.

These results justified the use of the baseline corrected Δ-SSQ ratings for further analyses. Although the Δ-SSQ ratings were positive, representing an increase in reported symptoms, in general, the increase was relatively low. The low ratings are not only attributable to the short VR exposure times but also to the fact that the previously set termination criterion systematically excluded four highly susceptible participants. Other rationales could include these particularly interesting participants by applying multiple imputation methods for data missing not at random [63]. Since the data are available in an open-access repository, the reader is welcome to reanalyze the data, taking the experimenter’s protocol into account for a closer estimation. Additionally, the generalizability is limited because the groups were not of equal sizes. Hence, the larger error in the smaller group—those starting with the RFT—increases error probabilities and contributes to the divergent findings. However, this finding cannot be attributed to a systematic exclusion of participants from this group, but rather to randomization that allotted slightly more participants to the City as the first application to which they were exposed.

As stated in the introductory section, especially individuals without any previous VR experience could profit from a screening instrument that would not require any history of cybersickness. Currently, this is a very relevant target group, since 60% of the current sample was inexperienced participants. However, no prediction of future states of cybersickness in another environment was possible with the RFT; rather, some indices—not all indices were equally indicative—were correlated with the current state for participants with instant previous VR experience (the group that took the RFT after the City). While the absolute deviation and the frame effect did not correlate with the Δ-SSQ_RFT_ to a significant extent, the frame influence as well as the constant error were significantly negatively correlated with the Δ-SSQ_RFT_. This—bearing the limitations of the performance measures in mind—invalidates the applicability of the RFT as a screening instrument [contrary to 64]. On the contrary, the RFT, especially the constant error and the field dependency index, could be used as an additional objective assessment of current well-being that functions almost language-free. Thus, it could be applied in studies aiming for higher inclusion, for example, recruiting younger or older participants, participants with hearing impairments, or whenever it is difficult to implement an oral assessment due to environmental factors. Further, using the RFT as an inference for current well-being would be advantageous, as reliance on self-reports for measuring cybersickness has been criticized [65]. However, these results require further replications to evaluate the robustness of the operationalization and the effect.

As field independence was only indicative of cybersickness in a specific case, it must be evaluated as a flexible construct [32]. As an explanation for the flexibility, the ability to reweight the mismatching information is noted [66]: According to the reweighting hypothesis, some individuals have difficulties inhibiting incongruent information, leading to higher awareness of a mismatch that manifests in cybersickness [32]. In line with the current results and sensory reweighting, earlier studies have found a shift towards field independence only for those participants, that had a “poorer” VR experience [32]. However, the direction of this effect would depend on the validity of visual, vestibular, and, proprioceptive information [67–70]. Thus, a good fit between the RFT and the application would be required, as expectations would be moderated by the context [29, applied examples from simulator sickness: 40, 68]. To validate this claim further evaluations in VR that include repeated RFT assessments are needed.

The RFT often deploys a seated position [28, 64, 71], while the VR applications often use interactive, standing VR [32, 64]. While historically, it makes sense for desktop applications and fixed-base simulators to test in a seated position, room-scale VR allows for higher comparability to the original room-filling physical apparatus [25]. The comparable setup in the current study should have addressed the same of proprioceptive and vestibular demands for an upright and stable stance in both environments [72]. It could be argued that the posture impacts RFT performance. Nevertheless previous studies suggest, that, unless a balance-challenging task is applied, the influence of the participant’s position (standing at ease vs. sitting down) appears to be negligible for the RFT performance [73].

In addition, to a potentially higher comparability of postural demands between the two VR applications a standing RFT setup enables an integrated repeated measure of field (in-)dependence during the VR exposure analogous to the MISC assessments. This way the stability of the RFT performance and the reweighting could be investigated in more detail while validating the applicability of a language-free assessment of the current state. Further, a repeated assessment during the VR exposure would make evaluations of the effectiveness of breaks obsolete. Nevertheless, the RFT assessment during the VR exposure would require shortening to be applicable. One solution could be an exclusion of one pair of the rod starting positions, which would result in 18 trials instead of 36.

Further, the current study contradicts previous results, that field dependent participants were likelier to experience cybersickness [64]. In contrast to the working mechanism presented in the introduction, field dependence is also hypothesized to be associated with a lower ability to ignore visual flaws [74, 75]. However, others report an advantage for field dependent participants in visual-proprioceptive-conflicting VR scenes, as they could ignore their physical position and suggested a higher sense of presence for field dependent participants [76]. What might integrate these contradictions is the conclusion that visual stimuli that are artificial, but are anticipatable lead to greater difficulties for field independent participants, because of the integration of proprioceptive input; while visual stimuli which are unanticipated are more provocative for field dependent participants, e.g., lags in frame rate [29]. Anticipation and controllability of movement can be estimated high in the current study, as no passive locomotion was deployed [77]. In addition, this study uses differing operationalizations in comparison to conflicting evidence, since field (in-)dependence was not only defined as the mean absolute deviation and cybersickness as the SSQ-Total Score post-VR. Neither the sole use of the absolute deviation nor the SSQ-Total Score is without criticism [20, 49, 78–80]. Therefore, a reanalysis of the previous studies using further indices could facilitate a mutual understanding of the differences between these diverging results.

Neither the absolute deviation nor the frame effect was significantly correlated with the Δ-SSQ. Both indices assume an equality for the clockwise and counterclockwise rotations, which is not supported by the current data. The current data indicate a high influence of the participant’s systematic response bias to one specific side. In more detail, the participants in general overcompensated to the right, which led to positive values of the deviation on the vertical axis. One explanation could be handedness [80, 81], which was not tracked in the Unity data or elicited in the descriptive questionnaire. In combination with the ambiguous results from the equivalence testing, future studies should focus on the validity of the RFT in comparison to concurrent field dependency measures [70, 75].

Additionally, while various reviews have discussed applicational and technical factors that contribute to cybersickness, while others are more user-centered [8, 9, 82–84]. However, their connections are less systematically evaluated. Although some studies focus on interconnections of, for example, posture and/or interaction possibilities in VR, there are only a few examples of systematically testing these in repeated measures designs [77, 85]. Little is known about the other contributing factors which were manipulated in the current study [e.g., sensed and awarded movement, visual complexity, exposure rationales; 9, 86, 87]

Since these factors might all contribute individually as well as in connection with each other, a one-size-fits-all screening method may be out of scope. Future research should address these different dimensions and their interconnection for the holistic VR experience. In order to enable context-sensitive replications, it is necessary to consistently pursue the open science concept. This includes not only open data and open methods but also open software for facilitated comparability and classification of divergent results.
